# Does stream flow structure woody riparian vegetation in subtropical catchments?

**DOI:** 10.1002/ece3.2249

**Published:** 2016-07-27

**Authors:** Cassandra S. James, Stephen J. Mackay, Angela H. Arthington, Samantha J. Capon, Anna Barnes, Ben Pearson

**Affiliations:** ^1^ Australian Rivers InstituteGriffith UniversityNathanQld4111Australia; ^2^ Centre for Tropical Water & Aquatic Ecosystem Research (TropWATER)James Cook UniversityTownsvilleQld4811Australia; ^3^ Department of Natural Resources and MinesWater ServicesPO Box 5318TownsvilleQld4810Australia; ^4^ Hydrobiology Pty LtdToowongQld4066Australia

**Keywords:** ELOHA, environmental flows, flood disturbance, hydrological modification, plant ecology, riparian forests, river management

## Abstract

The primary objective of this study was to test the relevance of hydrological classification and class differences to the characteristics of woody riparian vegetation in a subtropical landscape in Queensland, Australia. We followed classification procedures of the environmental flow framework ELOHA – Ecological Limits of Hydrologic Alteration. Riparian surveys at 44 sites distributed across five flow classes recorded 191 woody riparian species and 15, 500 individuals. There were differences among flow classes for riparian species richness, total abundance, and abundance of regenerating native trees and shrubs. There were also significant class differences in the occurrence of three common tree species, and 21 indicator species (mostly native taxa) further distinguished the vegetation characteristics of each flow class. We investigated the influence of key drivers of riparian vegetation structure (climate, depth to water table, stream‐specific power, substrate type, degree of hydrologic alteration, and land use) on riparian vegetation. Patterns were explained largely by climate, particularly annual rainfall and temperature. Strong covarying drivers (hydrology and climate) prevented us from isolating the independent influences of these drivers on riparian assemblage structure. The prevalence of species considered typically rheophytic in some flow classes implies a more substantial role for flow in these classes but needs further testing. No relationships were found between land use and riparian vegetation composition and structure. This study demonstrates the relevance of flow classification to the structure of riparian vegetation in a subtropical landscape, and the influence of covarying drivers on riparian patterns. Management of environmental flows to influence riparian vegetation assemblages would likely have most potential in sites dominated by rheophytic species where hydrological influences override other controls. In contrast, where vegetation assemblages are dominated by a diverse array of typical rainforest species, and other factors including broad‐scale climatic gradients and topographic variables have greater influence than hydrology, riparian vegetation is likely to be less responsive to environmental flow management.

## Introduction

The overriding influence of flow regime on riverine and riparian ecosystems has become a central axiom in freshwater ecology and the management of riverine systems (Poff et al. 1997; Bunn and Arthington 2002). Restoration efforts that aim to conserve and enhance riparian vegetation communities using flow manipulations (environmental flows) rely upon an understanding of relationships between flow and ecological responses (flow–ecology relationships). Yet there remain many thousands of riparian species and riverine systems for which this information is severely lacking (Arthington et al. 2006; Mackay et al. 2014). To address this information deficit, stream ecologists have proposed a new framework, the Ecological Limits of Hydrologic Alteration (ELOHA), designed to develop flow–ecology relationships for streams and rivers of contrasting hydrological character as determined by flow regime classification (Poff et al. 2010). The ELOHA framework is based on the premise that flow is a key determinant of the ecological characteristics of rivers and their riparian zones and that ecological character should therefore vary spatially in relation to the hydrological characteristics of distinctive flow regime classes (e.g., Mackay et al. 2014; Rolls and Arthington 2014). Ecological characteristics of river segments within each flow class are expected to be relatively similar and to differ from the ecological characteristics of river segments in other hydrological classes (Arthington et al. 2006). Accordingly, hydrological classes should be important in explaining variation in ecological patterns (McManamay et al. 2015). Patterns of similarity and difference in the ecological characteristics of hydrological classes have been the subject of recent studies (Chinnayakanahalli et al. 2011; Rolls and Arthington 2014; McManamay et al. 2015). However, the relevance of hydrological classification and class differences to a broader range of aquatic and riparian biota and landscape settings has not been tested.

Hydrology is widely recognised as the principal driver of riparian vegetation composition and structure throughout the world (Nilsson and Svedmark 2002; Naiman et al. 2005). Riparian species’ pools commonly reflect a combination of plant tolerances to the stresses imposed by surface water flow regimes, their capacity to capitalize on the subsidies provided by flow and their ability to regenerate after hydrologic disturbance (Naiman and Decamps 1997). Riparian vegetation assemblages vary mainly in relation to flow as well as geomorphology, which together largely determine patterns of water availability and fluvial disturbance (Merritt et al. 2010; Bendix and Stella 2013). Consequently, flow modification, such as that resulting from dams, is often associated with changes in riparian vegetation which, given its functional importance in the landscape, can have significant environmental and socioeconomic ramifications across multiple scales (Capon et al. 2013). Understanding the relevance of hydrological classification and class differences to the structure of riparian communities is potentially just as important to implementation of the ELOHA framework as studies focused entirely on in‐stream biota (e.g., fish, McManamay et al. 2012; Rolls and Arthington 2014; McManamay et al. 2015).

Although hydrological classification and the influence of class differences on flow–ecology relationships form the main platform of the ELOHA framework, the “hydrological foundation” can be extended to include a geomorphic subclassification as a further means to understand the main environmental influences on riverine and riparian communities (Poff et al. 2010). Furthermore, other environmental factors (e.g., climatic variables such as temperature and rainfall, topography, physical channel characteristics, hydraulic conditions, and substrate type) and the catchment context may also influence variation in riparian vegetation. The information gained by incorporating potentially confounding variables into flow–ecology relationships is not part of the ELOHA framework, but is beginning to be explored in recent studies (e.g., McManamay et al. 2013; Arthington et al. 2014).

The catchment context may be particularly important because environmental flow management frequently occurs in highly modified agricultural and urban regions, where riparian vegetation frequently represents the sole native vegetation remaining in the landscape (Maisonneuve and Rioux 2001). Often persisting as thin and fragmented strips, riparian vegetation within such landscapes is likely to be vulnerable to a wide range of anthropogenic pressures associated with the direct effects of human activities in the riparian zone (e.g., clearing, grazing, cropping, and burning) as well as indirect effects of land uses in surrounding catchments (e.g., pollution from agrochemicals, invasion by pastoral species, and changes to runoff and sediment transport patterns; Richardson et al. 2007; Bowers and Boutin 2008). Such pressures can result in greater cover of exotic species, reduced native species diversity, altered plant density, or disrupted successional patterns (e.g., Johnson 1999; Corbacho et al. 2003; Aguiar and Ferreira 2005; Lopez et al. 2006; Bruno et al. 2014). The role of flow in structuring riparian vegetation in highly modified landscapes may therefore be masked, diminished, or amplified by many anthropogenic pressures and their effects. Nevertheless, environmental flow management has the potential to promote improvements in riparian condition and river health even where land use has an overwhelming influence on riparian ecology (Johnson 1999). If the ELOHA framework is to have broad‐scale applicability, its utility and the assumptions that underpin it need to be tested within modified landscapes to guide decision‐making. Studies of flow–ecology relationships rarely address the influence of catchment modification and land use change (Stewart‐Koster et al. 2010; Arthington et al. 2014). Indeed, in many cases, study designs actively avoid potentially confounding effects of land use on flow regimes and riparian/aquatic ecosystems.

The primary objective of this study was to test the relevance of hydrological classification and class differences to the characteristics of woody riparian vegetation in streams of subtropical southeast Queensland. The study of riparian communities formed part of a regional trial of the ELOHA framework (see Arthington et al. 2012). To address the primary study objective, we used two approaches. Firstly, we examined how riparian vegetation metrics and assemblages varied across a flow classification for the southeast Queensland region (Mackay et al. 2014). We selected riparian metrics describing species diversity, successional stage, exotic status, and regeneration as these metrics describe key characteristics of riparian vegetation and have tangible links to flow and flow regime change (Nilsson and Svedmark 2002). Secondly, we investigated to what extent any observed riparian patterns among the hydrological classes could be attributed to other drivers of riparian assemblage structure that may covary with flow class. We selected additional variables describing the physical environment (climate, rainfall, topography, hydraulic conditions, and substrate type) and two pressures (land use and degree of hydrological alteration) because of their potential to influence riparian vegetation. Riparian zones of subtropical catchments are highly dynamic and prone to fluvial disturbance because of their strongly seasonal stream flows and the occurrence of intense rainfall events during the year. We therefore included a descriptor of fluvial disturbance (specific stream power) reasoning that this is likely to be a key mechanism via which flow influences riparian vegetation in the region. Degree of hydrological alteration was included as a potential covariant to take account of the fact that one hydrological class was composed mainly of streams with flow patterns modified by upstream dams, and most streams we studied had experienced minor changes in some flow characteristics compared to modeled predevelopment flows.

## Methods

### Study area

**Figure 1:**
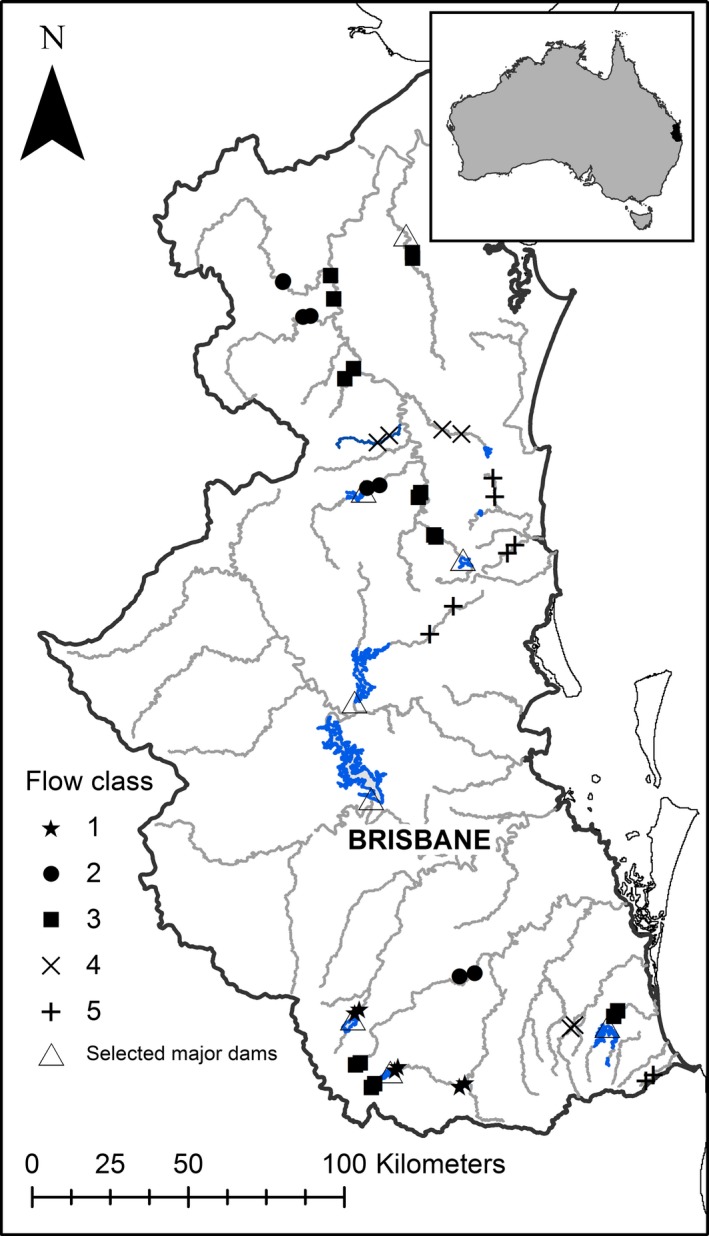
The southeast Queensland study region and site locations. Grey lines are major rivers and the solid black line is the study extent. Blue areas are large dams.

The study was conducted in southeast Queensland, Australia (Fig. 1). Climatically, the region is subhumid and subtropical. Rainfall occurs throughout the year but declines strongly along an east–west gradient with mean annual rainfall ranging from 1400 mm on the coast to 800 mm inland (Bridges et al. 1990). The region comprises seven major river catchments. Higher mean annual runoff per unit area occurs in the eastern coastal catchments. Due to the irregularity of rainfall across the region, flow regimes in all of the region's rivers and streams are highly variable but generally have late summer–early autumn high discharge regimes, with periods of low discharge and intermittent zero flows occurring from August to November (Pusey et al. 2004).

The region exhibits considerable topographic and geomorphologic variation and is associated with complex geology and soils. Distinct topographic areas in the region include coastal plains, river floodplains, and large estuaries in the east and foothills and mountains with plateaux over 300 m a.s.l. to the west, north, and south. Land use in the region is predominantly agricultural (~60% of land area), mainly comprising grazing on relatively natural pastures. Much of the region has also been extensively cleared of native vegetation, mostly by the 1940s, but with clearing continuing in recent decades (Bradshaw 2012).

There are 24 dams with crest heights over 15 m in the study region, most of which were constructed in the early 1970s to mid‐1970s to support irrigated agriculture and urban development. Although dams have had significant effects on downstream flow regimes, many predevelopment hydrological characteristics persist in the streams of the region (Mackay et al. 2014). The main changes to flow regimes from predevelopment conditions include a loss of natural flow diversity and an increase in the duration of low flow spells (Mackay et al. 2014).

### Site selection

Sites were selected as part of a regional trial of the ELOHA framework that also considered flow–ecology relationships for fish and aquatic vegetation (Arthington et al. 2012, 2014; Mackay et al. 2014). To provide the “hydrological foundation” of this trial, a classification of historic flow regimes was conducted using stream gauge data with an average of 25 years of flow record and a minimum of 15 years of record within the period 1975–2000. This analysis and the terminology used to describe the different flow classes (Table 1) are based on a national hydrologic classification (Kennard et al. 2010). The analysis of Mackay et al. (2014) identified five flow classes across the region (Table 1). Classification was undertaken using model‐based hierarchical agglomerative clustering based on Gaussian finite mixture models, as implemented in the Mclust package for R (R Core Development Team 2010). Flow classes were distinguished from each other mainly by hydrologic metrics associated with flow magnitude, duration of high‐flow pulses, number of zero‐flow days, and the constancy of mean daily flow (calculated using Colwell's index of constancy Colwell 1974) (details of the flow classification procedure are provided in Mackay et al. 2014). In broad terms, these classes can be described as perennial (class 1), rarely intermittent (class 3), intermittent (class 4, 5), and highly intermittent (class 2). Flow classes 2–5 reflect similar “reference” flow classes developed from modeled predevelopment hydrologic data, while flow class 1 is an artificial flow class reflecting river regulation by dams and other flow modifications (Table 1; Mackay et al. 2014).

**Table 1:** Descriptions of flow regimes characterizing flow classes for rivers of subtropical southeast Queensland (adapted from Rolls and Arthington 2014). Values in brackets indicate the number of sites in each class

Flow class	Description of flow regime
1 (6)	*Perennial* (artificially); high–minimum flow, low flood magnitude, short duration of high‐flow pulses, no zero‐flow days, high constancy of flow
2 (8)	*Highly intermittent‐unpredictable summer*; low–minimum flow, low flood magnitude, long duration of high‐flow pulses, high proportion of zero‐flow days, and moderate constancy of flow
3 (16)	*Rarely intermittent‐unpredictable*; moderate–minimum flow, moderate flood magnitude, moderate duration of high‐flow pulses, low proportion of zero‐flow days, and low constancy of flow
4 (6)	*Unpredictable*; low–minimum flow, high magnitude of large floods, moderate duration of high‐flow pulses, high proportion of zero‐flow days, and low constancy of flow
5 (8)	*Intermittent‐unpredictable;* moderate–minimum flow, high magnitude of large floods, short duration of high‐flow pulses, low proportion of zero‐flow days, and low constancy of flow

We selected 44 sites, spread across the five flow classes, based on proximity to stream gauges, accessibility, and limited direct modification of riparian vegetation from activities such as clearing, burning, and/or grazing (Fig. 1). All sites were positioned in mid‐ and lowland stream reaches of <300 m elevation, and regulated and nonregulated reaches were included. While we excluded sites that had been directly impacted by clearing in the last 20–30 years, all of our sites had more than 24% (and up to 92%) of their catchment area subject to agricultural activity. To minimize within‐site variation in stream morphology, geology, and adjacent land use, but still include multiple in‐stream habitats, field sites comprised 100 m long stream reaches.

### Data collection

#### Vegetation survey

We surveyed woody riparian vegetation in three randomly positioned 5‐m‐wide belt transects at each site. All transects were located on the same bank so that land use impacts were similar within a site. Up to three additional transects were surveyed at three sites due to very low plant densities (i.e., <100 individuals) in initial transects. Transects extended up to 70 m from the water's edge to the edge of the riparian vegetation. In the few cases where land use change did not occur within 50 m of the water's edge, landform change (e.g., a distinct change in bank slope) was used to delineate the upland extent of each transect. Transects ranged from 12.5 to 69 m with a median length of 32.4 m. We recorded the presence and diameter at breast height (dbh) of all trees and shrubs >50 cm tall within each belt transect. Field work was undertaken between 2008 and 2010.

#### Environmental variables

To characterize the substrate at each site, we collected soil samples along each transect at the stream edge, midway along the transect, and bankfull (i.e., the point at which water begins to overflow onto a floodplain or surrounding landscape; Rosgen 1996). Additional soil samples were taken where cross‐sections intersected other distinct landforms (e.g., benches and bars), although these were not common at the selected sites. A hydrometer was used to determine the proportion of silt, clay, and sand in each sample, and mean values were calculated for the site. Mean annual temperature and mean annual rainfall for each site were determined from national datasets (Bureau of Meteorology 2009; Stein et al. 2009).

We used bank height above the waterline, measured via two or three cross‐sectional surveys with an optical level at each site, as a proxy for depth to the water table because groundwater can have a significant influence on riparian vegetation assemblages. To characterize the degree of fluvial disturbance and therefore the potential for mechanical damage to plants (Bendix 1999), we also calculated stream‐specific power (SSP, W/m^2^) for each site using the formula SSP = yQS/w, where y is the unit weight of water (9800 N/m^3^), Q is discharge (m^3^/sec), S is energy slope (m/m) approximated by bed slope, and w is channel bankfull width (m) (Bizzi and Lerner 2015). We used the 2‐year annual return interval (ARI) floods as the reference discharge as this is approximately equal to bankfull discharge (Wharton 1995). The 2‐year ARI floods may not reach all the riparian vegetation at every site; however, this ARI is highly correlated with larger flood magnitudes (i.e., correlation between 2‐year and 10‐year ARI > 0.99) and it is therefore unlikely to affect the overall results. Stream slope and channel bankfull width were obtained from field surveys using an optical level and stave except for three sites for which channel slope was estimated from 25,000 scale mapping due to constraints accessing sites.

We determined the proportion of each site's catchment under agriculture using land use data from the Queensland Land Use Mapping Program, generated from 1999 baseline surveys (Witte et al. 2006). Draft updates available from 2006 land use surveys for the Maroochy and Logan–Albert were also incorporated (DERM 2010). Because land use closer to streams may have a disproportionate influence on stream condition relative to distal land uses, we calculated an inverse‐distance weighting (*d + *1)^−1^ metric following Peterson et al. (2011).

We also determined the degree of hydrologic alteration for each site, represented by a Gower dissimilarity metric which was based on the difference between modeled predevelopment (i.e., natural) flows and historic (gauged) flows as described by Mackay et al. (2014). Modeled, natural flow data were unavailable for four sites but as none of these were regulated, flow modification at these sties was assumed to be similar to nearby nonregulated streams.

### Data analysis

We calculated vegetation metrics based on the cumulative survey data for each site (Table 2). We determined species richness and abundance per hectare (ha) (Table 2). Each species was assigned a successional stage characterized as early (E), intermediate (M), or late (L) and combinations of these stages, that is, where the species occurred in more than one successional stage, as EM, ML, or EML, following Kanowski et al. (2010). Proportions of individuals classified as early, intermediate, and late successional stages were determined as a percentage of the total abundance. Because many species are classified as combinations of these stages, the total percentage of early, intermediate, and late can sum to more than 100%. Proportions of exotic trees and shrubs were determined for each site. Trees with a dbh ≤ 10 cm were all categorized as regenerating (Kariuki and Kooyman 2005). Vegetation metrics and assemblage data were calculated for both the whole transect length, hereafter “bankfull”, and for the “near‐stream” zone which included the transect area <5 m from the water's edge because regional floristic surveys suggest that is where most rheophytic species are confined.

**Table 2:** Metrics describing characteristics of woody riparian vegetation in streams of subtropical southeast Queensland

Metric	Description
SPECIES RICHNESS	Species richness per ha
ABUNDANCE	Abundance of trees and shrubs per ha
EARLYPER	Proportion of early successional species
INTERPER	Proportion of intermediate successional species
LATEPER	Proportion of late successional species
EXOTICPER	Proportion of exotic trees and shrubs
NATIVE REGEN	Abundance of native regenerating (DBH <10 cm) trees and shrubs per ha
BASAL AREA	Basal area of trees and shrubs per ha

We used Kruskal–Wallis tests followed by multiple post hoc comparison tests using the kruskalmc function of the pgirmess package in R which implements the method of Siegel and Castellan (1988) to examine differences in vegetation metrics and common species’ abundances (i.e., species occurring at more than 20 sites) across flow classes.

To assess the effects of hydrological class on vegetation assemblages, we conducted permutational multivariate analysis of variance using Bray–Curtis distance matrices with the Adonis function in R's “vegan” package (Oksanen et al. 2010). Prior to this analysis, species abundance data were log10(*x* + 1)‐transformed and rare species (i.e., those occurring in less than three sites) removed. Permutational multivariate analysis of variance is sensitive to heterogeneity in dispersion particularly for unbalanced designs (Anderson and Walsh 2013) so we explored differences in dispersion between flow classes using the PERMDISP test for homogeneity of dispersions (Anderson 2006). We also calculated the indicator values of species for each flow class as the product of the relative frequency and relative average abundance in flow classes. Indicator value is maximized (i.e., 1) when all individuals of a species are found in a single flow class (high fidelity) and when the species occurs in all sites in that class (high constancy). This analysis was conducted using Dufrene–Legendre indicator species analysis with the indval function in the “Labdsv” package of R. Differences in assemblages across flow classes were visualized using nonmetric multidimensional scaling (nMDS) based on Bray–Curtis distance matrices in the “Vegan” package of R.

We explored relationships between vegetation metrics and the nonflow environmental variables using the glmulti package in R (Calcagno and de Mazancourt 2010). This approach uses linear modeling to fit all possible models which are then compared using Akaike's information criterion (AICc) adjusted for small sample size. We restricted our model selection to main effects only, due to the relatively small number of sites, and compared models using the AICc criterion. We discarded all models with parameter coefficients that were not found to be different from zero (*P *<* *0.05). We assumed a Gaussian distribution for all response variables apart from proportional data (successional stages and exotics as a proportion of total individuals) for which we used the binomial family with a logit link function. All data sets were checked to make sure they fitted the assumptions of linear models and transformed where necessary. Environmental predictors were standardized. Square root transformation was applied to species richness, the total abundance of trees and shrubs, the abundance of regenerating native, and the basal area of trees and shrubs. Variance inflation factors (VIF) were employed to check for model fit and collinearity among the predictors. As none of the VIF's inspected exceeded 2, the standardized regression coefficients were assumed to be reliable estimates.

To explore relationships between environmental variables and assemblage structure, we first used the BIO‐ENV procedure in the “Vegan” package in R to identify the best subset of environmental variables, including flow class that minimized the Gower distances of scaled environmental variables to have the maximum rank correlation with the community dissimilarity matrix (Clarke and Ainsworth 1993). Gower distances were used because the environmental variables included both quantitative and categorical predictors (i.e., flow class). We then used redundancy analysis (RDA) implemented with the “varpart” function in R to partition variance in assemblage composition explained by flow and nonflow environmental metrics. This is a constrained ordination method which does not attempt to explain all the variation but only the part that can be explained by the used constraints (parameters). We grouped climate variables (annual rainfall and mean annual temperature), hydrological variables (flow class, specific stream power, degree of hydrological alteration, and bank height as a surrogate for depth to the water table), and land use (distance‐weighted proportion of modified land use) for this analysis. We applied a Hellinger transformation following Legendre and Gallagher (2001) that allows data that have nonlinear response to be analyzed using RDA and also allows the use of adjusted R values to account for the different numbers of variables in the environmental predictor groups (Peres‐Neto et al. 2006). All analysis was performed in R version 3.1.1 (R Development Core Team 2010).

## Results

Over 15,500 trees and shrubs were identified and recorded across the 44 survey sites representing 191 tree and shrub species (Table S1). The most diverse sites on Currumbin Creek (the southern limit of the study area and adjacent to the New South Wales border), Amamoor Creek and Yabba Creek (the Mary River catchment), and the Stanley River (western headwaters of the Brisbane catchment) had 49, 45, 45, and 44 tree and shrub species, respectively. The most abundant native species were *Ficus coronata* (Sandpaper Fig), *Castanospermum australe* (Black Bean), *Cryptocarya triplinervis* (Three Veined Laurel), and *Syzygium floribundum* (Weeping Lilly Pilly). Exotic taxa comprised 26.5% of all individuals recorded. The most abundant exotic species were *Celtis sinensis* (Chinese Elm), *Lantana camara* (Lantana), *Leucaena leucocephala* (Leucaena), *Cinnamomum camphora* (Camphor Laurel), and *Ligustrum lucidum* (Broad‐leaved Privet). Densities of trees and shrubs per ha ranged from just under 1000 trees and shrubs per ha (Burnett Creek site 27) to over 21,500 (Teviot Brook). The extremely high tree and shrub density recorded at Teviot Brook was due to a very large number of *Celtis sinensis* recruits, hence this site also had the highest density of exotic tree regeneration (>20,000 per ha). Proportions of trees belonging to the different successional stages (E, M, L) varied considerably across the sites. Overall, early successional stage species comprised around 24% of all individuals recorded, while intermediate and late successional stage species comprised 42% and 33%, respectively.

### Relationships between flow class and vegetation

Significant differences across flow classes were detected for species richness, abundance, and abundance of regenerating native trees and shrubs (Figure S1). No significant pairwise differences were detected at the *P *<* *0.005 significance level (a conservative adjusted *P* value to take into account of the multiple comparisons); however, there were pairwise differences at the *P *<* *0.05 level in species richness and abundance. Sites in flow class 5 had higher species richness and abundance than sites in flow class 1, and higher species richness than sites in flow class 2. Significant differences between flow classes were also detected in the distribution of three common tree species; *Melaleuca viminalis*,* Casuarina cunninghamiana,* and *Streblus brunonianus* (Figure S2). *C. cunninghamiana* was relatively common in flow class 1 (artificial perennial class containing regulated streams, Table 1) but virtually absent from flow class 5 (intermittent, unpredictable flows, Table 1), while *S. brunonianus* was common in flow class 3 (rarely intermittent, unpredictable flows, Table 1) but relatively uncommon in both flow class 1 and 5. Significant pairwise differences were only found, however, for *M. viminalis* between flow classes 2 and 5 with this species significantly less common in the latter class (*P *<* *0.005). No significant differences were found across flow classes for proportion of different successional stages, proportion of woody exotic species, or basal area.

**Figure 2:**
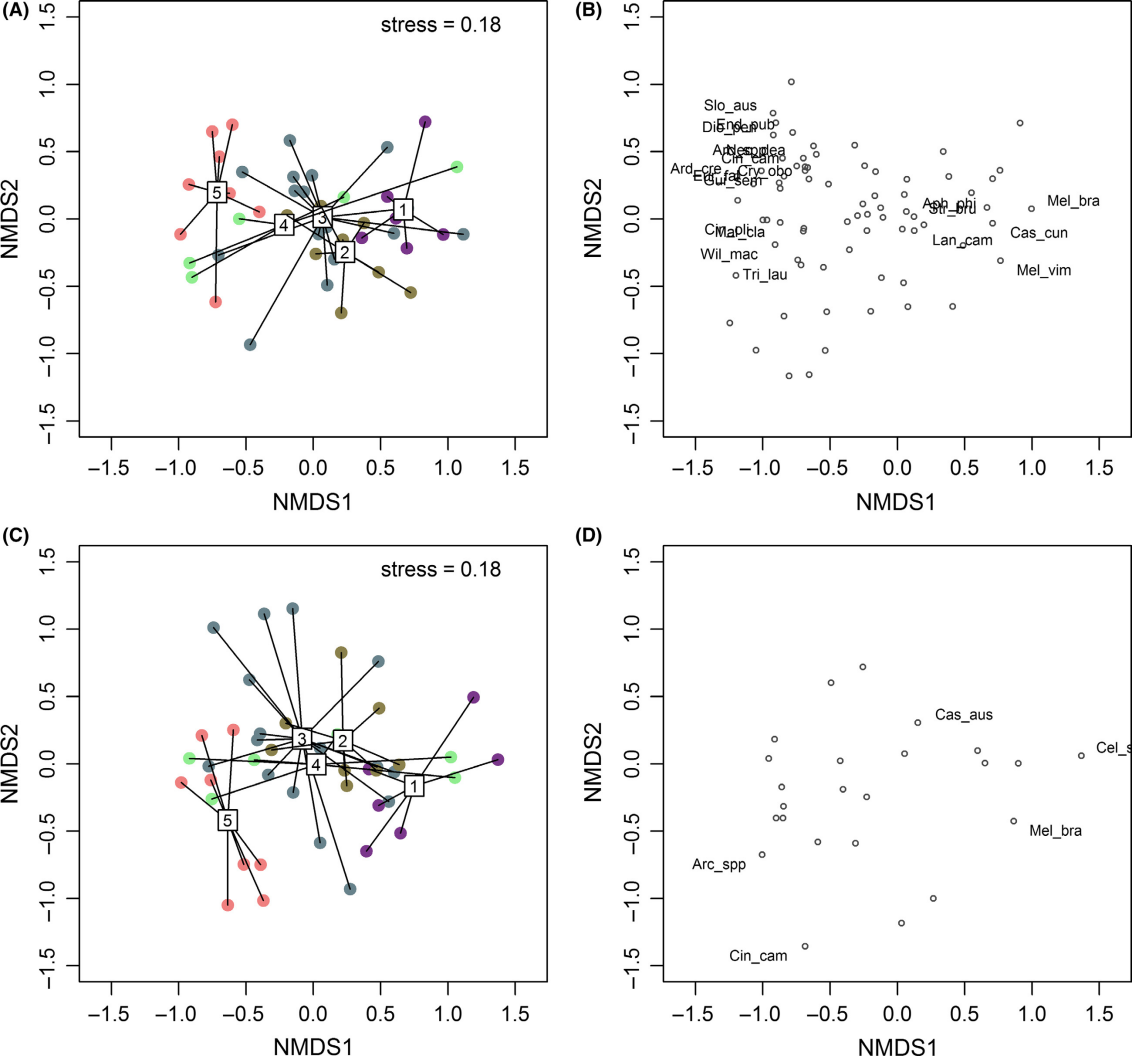
Nonmetric MDS ordination of sites based on log(*x + 1)*‐transformed tree and shrub assemblage data, two dimensions. (A) Position of sites and flow classes in ordination space for bankfull vegetation, (B) species identified through indicator species analysis as having high habitat fidelity and specificity for the flow classes from the bankfull vegetation dataset, (C) position of sites and flow classes in ordination space for near‐bank vegetation, (D) species identified through the indicator species analysis as having high habitat fidelity and specificity for the flow classes from the near‐bank vegetation dataset. Species codes are given in Table S2.

Based on the permutational multivariate analysis of variance, an effect of flow class was detected for both bankfull (*F*_4,39_ = 3.66, *P *=* *0.001) and near‐stream (*F*_4,39_ = 2.63, *P *=* *0.001) vegetation assemblages. This was also evident in the nMDS ordination (Fig. 2) where sites belonging to flow classes 1 and 5 were generally well separated in ordination space. Sites in flow classes 2, 3, and 4, however, were less well separated and showed a high degree of overlap. Site dispersion within each flow class did not differ significantly between classes, suggesting that the vegetation differences detected between them were due to means rather than within‐class variation. Twenty‐one indicator species, mostly native taxa, distinguished between vegetation assemblages of each flow class (Fig. 2; Table S2). Most of these indicator species were associated with flow class 5, four species including one exotic species, *Celtis sinensis,* were associated with flow class 1 (artificial flow class), two native species and one exotic species, *Lantana camara,* were associated with flow class 2, and one species was associated with flow class 3 (*Streblus brunonianus*).

### Do other environmental variables vary across flow classes?

Other environmental variables were found to vary significantly across flow classes (i.e., annual rainfall, mean annual temperature, sand, clay, stream‐specific power (SSP), and land use modification; Figure S3). At the *P *<* *0.005 significance level (a conservative adjusted *P* value to take into account of the multiple comparisons), there were pairwise differences across flow classes for annual rainfall, mean annual temperature, proportion of clay in substrate, and SSP. Annual rainfall was significantly higher for sites in flow class 5 compared with sites in flow classes 1 or 2. Proportion of clay was significantly higher in flow class 5 compared with flow class 2 and SSP significantly higher in flow class 3 compared with flow class 2. No significant differences across flow classes were found for bank height (a proxy for depth to the water table) or the Gower metric (as a measure of overall flow modification), although the latter tended to be higher for sites in flow class 1 (the class containing mostly regulated sites) compared to the other flow classes.

### Relationships between nonflow environmental variables and vegetation

Linear models with significant parameter coefficients were identified for four of the riparian metrics and their near‐stream counterparts using an exhaustive search of all main effects; species richness, abundance, regeneration of native species, and basal area. All but one of the best models returned included annual rainfall with a significant coefficient (Table 3). Annual temperature was also significant for species richness and abundance of native regeneration, while bank height and proportion of clay were only significant for species richness and basal area of near‐stream trees and shrubs, respectively (Table 3). None of the remaining environmental variables (i.e., proportion of sand, stream‐specific power, land use, or flow modification) were significant parameters in the returned models.

**Table 3:** Results of linear models showing significant parameters in the best models for riparian metrics for full bank and near‐stream (with suffix NS) vegetation of subtropical southeast Queensland

Riparian metric	Mean annual rainfall		Mean annual temperature		Bank height		Proportion of clay	
	Estimate	SE	Estimate	SE	Estimate	SE	Estimate	SE
SPECIES RICHNESS	0.0347***	0.009	0.0238**	0.0085	−0.0178	0.009		
ABUNDANCE	12.933**	3.824						
NATIVE REGEN	13.904***	3.314	6.708**	3.824				
BASAL AREA	0.631*	0.299						
SPECIES RICHNESS NS	0.047***	0.012						
NATIVE REGEN NS	10.207*	4.020						
BASAL AREA NS							0.199**	0.068

***P <* *0.001, **P <* *0.01, *P <* *0.05.

The BIO‐ENV analyses indicated that best subset of predictors explaining variation in riparian vegetation assemblages for both the near‐stream and bankfull riparian vegetation assemblages was a combination of annual temperature and annual rainfall (correlations of 0.47 and 0.36 with bankfull and near‐stream vegetation assemblages, respectively). Both bankfull and near‐stream vegetation assemblages were most strongly correlated with annual rainfall, which varied strongly between flow classes along the first ordination axis. Annual temperature also correlated significantly with ordinations of vegetation assemblages and was generally lower in flow class 1 compared with the other flow classes. Redundancy analysis with variance partitioning was then used to assess the variation in assemblage structure explained independently and shared by climate, hydrology, and land use variables (Table 4). For the bankfull data set, the parameters selected explained 15% of the vegetation assemblage pattern, while for the near‐stream data set, the parameters selected for modeling explained less (12%) (Table 4). This proportion rose slightly to 16% when only common species were analyzed. For all partitioning, the climate variables independently explained more variation than the hydrology variables independently (Table 4). Land use variables did not explain any of the variation in assemblage structure. The variation shared between hydrology and climate was greater than that of any of the variable groups independently.

**Table 4:** Partitioning of variation in redundancy analysis for different riparian vegetation data sets. Given are the adjusted R squared for the testable fraction, the df (degrees of freedom) and the *F*‐ and *P*‐values for all full bank data set, the near‐bank dataset, and abundant species (those species occurring in 20 or more sites) in the full bank data set. Fraction [Hydro] = variation dependent upon hydrological variables alone; fraction [Climate] variation dependent upon the climate variables alone; fraction [Land] variation dependent upon land use modification alone; fraction [Hydro+Climate] variation shared between hydrology and climate; fraction [Hydro+Land] variation shared between hydrology and land use; fraction [Climate+Land] variation shared between climate and land use

Data set	Total explained variation	Hydro	Climate	Land	Hydro+ Climate	Hydro+Land	Climate+Land	df	*F*	*P*
Bank full	0.1474	0.0196	0.0502	−0.0005	0.0786	0.0070	−0.0007	10	1.7432	0.001
Near stream	0.1193	0.0148	0.0391	−0.0009	0.0645	0.0004	−0.0002	10	1.5823	0.001
Abundant species	0.1642	0.0034	0.0763	−0.0075	0.0982	0.0023	−0.0100	10	1.8449	0.001

## Discussion

Hydrological classifications underpin recent environmental flow initiatives such as ELOHA (Poff et al. 2010), consequently ascertaining the relevance of hydrological classes to critical aquatic and riparian communities must be a priority. Patterns of similarity and difference among hydrological classes have been reported for fish and invertebrates (Chinnayakanahalli et al. 2011; Rolls and Arthington 2014; McManamay et al. 2015); yet to our knowledge, the relevance of hydrological classification and class differences to riparian vegetation communities has not been tested.

Riparian vegetation of subtropical southeast Queensland associated with drier, inland streams was characterized by a relatively small suite of species, whereas rainforest sites, which were particularly prevalent along coastal creeks and in the northern Mary River catchment (Fig. 1), were typified by a diverse assemblage of rainforest species, including many not usually considered to be obligate riparian plants. Many of the most common species recorded in the riparian zones (e.g., *Cryptocarya triplinervis*) are not usually considered rheophytic and occur across a range of terrestrial habitats, suggesting factors other than flow are likely to be significant in determining their distributions and abundance.

In southeast Queensland, differences in riparian characteristics among classes were evident for riparian species richness, abundance, and abundance of regenerating native trees and shrubs. There were also significant differences in the occurrence of three common tree species; *Melaleuca viminalis*,* Casuarina cunninghamiana,* and *Streblus brunonianus* between flow classes. An effect of flow class was detected for both bankfull and near‐stream vegetation assemblages, and 21 indicator species (mostly native taxa) distinguished between vegetation assemblages of each flow class. These findings suggest that variations in stream flow across the major coastal‐inland, wet‐dry gradient of our study area influenced the distribution, richness, and composition of riparian vegetation. However, the variation between flow classes in structural vegetation metrics and overall vegetation assemblages could also be explained by nonflow metrics that varied across flow classes. Climate, particularly annual rainfall which covaried strongly with flow class, was found to be a significant parameter in best models for species richness, vegetation abundance, native woody vegetation regeneration, and basal area as well as independently explaining a higher proportion of variation in assemblage structure relative to hydrology or land use. Mean annual temperature also emerged as a significant driver of overall riparian vegetation assemblages, species richness, and native regeneration, further emphasizing the critical influence of regional climatic variation on riparian vegetation. Because of the strong covariation between rainfall and hydrological class, it is not possible to draw any strong conclusions regarding the relative importance of flow versus climate. However, the prevalence of rheophytic species in some flow classes (e.g., 1 and 2) and their virtual absence from other flow classes (flow class 5) implies a comparatively greater role for hydrology in the former which needs further testing.

**Figure 3:**
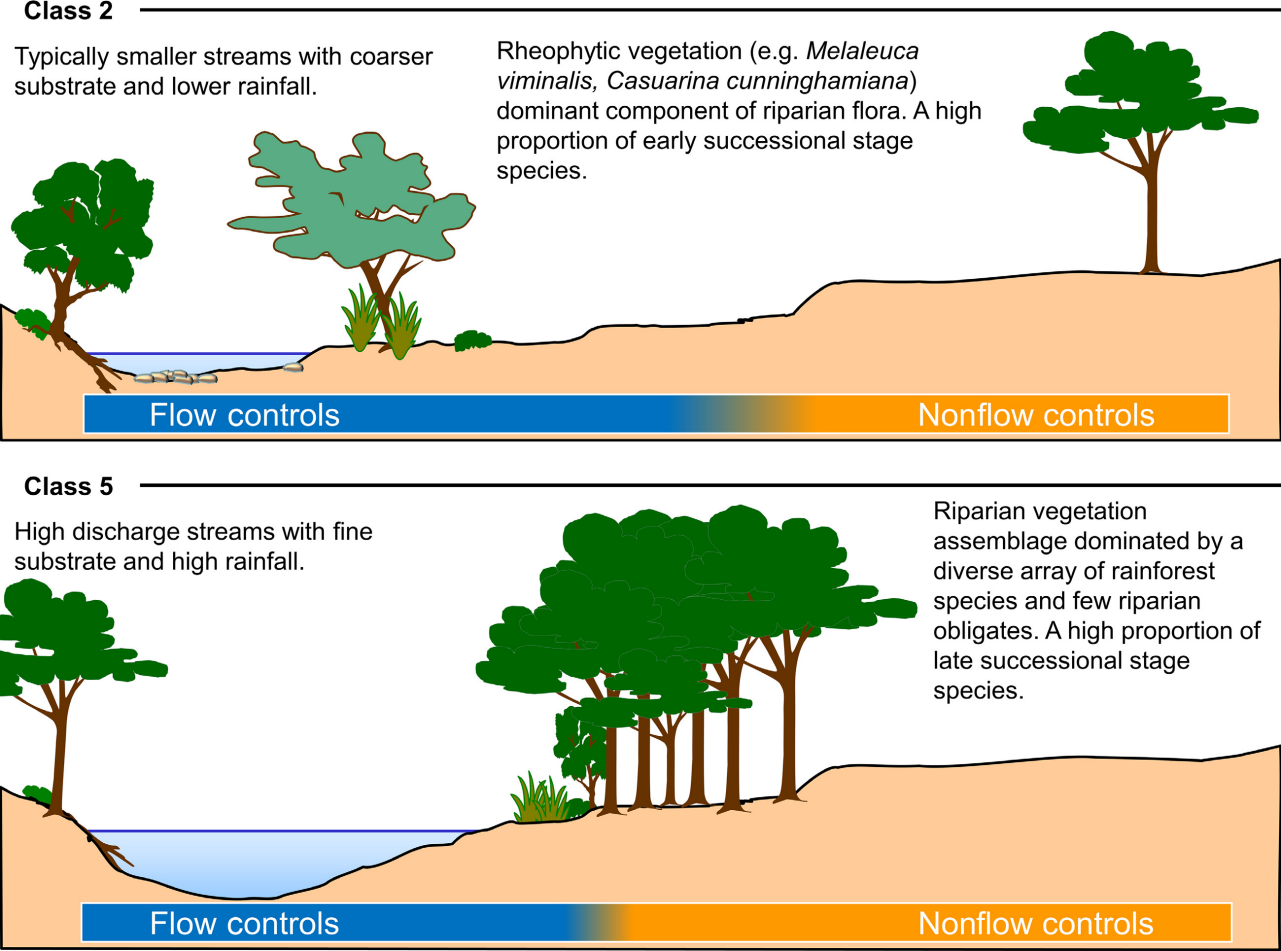
Conceptual diagram contrasting streams in flow class 2 (highly intermittent–unpredictable summer; Table 1) and flow class 5 (intermittent–unpredictable; Table 1) and their associated riparian vegetation. Under this framework, management of stream flows to influence riparian vegetation would have most potential for streams in flow class 2.

In Figure 3, we provide a conceptualization of the relative influences of flow versus other broad‐scale drivers (e.g., climate) using sites from two contrasting flow classes; class 2 and class 5 (Table 1). Sites in flow class 2 are typically smaller streams with coarser substrates and lower rainfall than those in flow class 5 which are high discharge streams with typically clay‐dominated substrates and high rainfall. The vegetation in these groups also contrasts strongly with rheophytic species tending to be a more dominant component of the vegetation of sites in flow class 2 (Fig. 3). Under this framework, management of flows to influence riparian vegetation assemblages would have most potential in those sites for which hydrological influences override other controls, such as those in flow class 2. In contrast, for sites in flow class 5, with vegetation assemblages dominated by a diverse array of typical rainforest species, other factors including broad‐scale climatic gradients and topographic variables are likely to have greater influence.

Published analyses of riparian vegetation distribution patterns highlight the importance of broad‐scale predictors such as climate, geology, and soils in providing the overriding controls on the distribution of riparian vegetation at a broader landscape scale (e.g., Tabacchi et al. 1996; Dixon et al. 2002; Sarr and Hibbs 2007). Climate has a major influence on stream flow and on hydrological class membership (Chinnayakanahalli et al. 2011) and riparian vegetation (e.g., Alcaraz et al. 1997; Sarr and Hibbs 2007). The observed distribution patterns of riparian vegetation in southeast Queensland are consistent with these broad‐scale influences of climate and hydrology; however, we also considered the influence of local environmental factors on riparian ecology.

At the local scale, riparian vegetation distribution patterns are typically zoned laterally along transverse gradients with distance and height from the stream edge. These lateral vegetation distribution patterns reflect the relative tolerances of species to physical disturbances such as shear stresses associated with stream hydraulic conditions and chemical stresses (anoxia and chemical toxicities) associated with water‐logged soils adjacent to streams and, the ability of species to acquire or intercept resources such as moisture (Lite et al. 2005), light (Hall 1998; Battaglia and Sharitz 2006), and nutrients (Kotowski et al. 2006) at different positions along the lateral gradient. Prior to this study, we hypothesized that fluvial disturbance is likely to be the main mechanism via which flow influences riparian vegetation in subtropical catchments because riparian zones in these landscapes are highly dynamic and prone to fluvial disturbance due to their strongly seasonal stream flows and the occurrence of intense rainfall events at various times during the year. Conversely, effects of flow on moisture provision to riparian vegetation may be less important in subtropical catchments, where water is more plentiful throughout the year than it is in some other climatic regions. Our measure of fluvial disturbance (specific stream power), however, was not related to either bankfull nor near‐stream vegetation assemblages. Furthermore, no relationship with specific stream power was found for any of the selected riparian metrics.

Studies in other climatic regions have also failed to show effects of fluvial disturbance on woody vegetation metrics (e.g., Lite et al. 2005), despite the strong conceptual basis for assuming such relationships. Descriptions of stream power determination in the ecological literature, however, often belie the practical issues around estimating this parameter. Stream power estimates are highly sensitive to the energy slope and the distance over which this is calculated (Barker et al. 2009). It is possible that our field estimates of stream power based on local field slopes (determined over <600 m stream lengths) may be capturing too much local variation rather than the scale likely to influence riparian habitats (Jain et al. 2006).

Stronger relationships between flow and riparian vegetation assemblages in southeast Queensland may be masked by flow modification and lagged responses of the vegetation to this disturbance. Many of the species recorded in our survey are relatively long‐lived (i.e., >100 years). Consequently, their current distribution and abundance may reflect flow conditions prior to river regulation and the period for which historical gauge records were available to develop flow classes (i.e., post 1975; Mackay et al. 2014). Although we included a measure of flow modification in our analyses, this Gower metric represents the full suite of changes in flow attributes that have occurred while, individually, these changes varied in relation to the type of dam, its location, and its operational patterns (Mackay et al. 2014). In other words, the same Gower estimate can result from alterations in different flow attributes which may in turn trigger contrasting vegetation responses.

Somewhat surprisingly, given that our study area has experienced significant land use change over the period of human settlement and agricultural development (Bradshaw 2012), we did not detect any effect on woody riparian vegetation composition or structure of agricultural land use intensity (measured as proportional area) in the surrounding catchment. As all of the sites had a relatively large proportion of their catchment under agriculture (i.e., mostly >45% with only four sites having <45% of land use modified), it is possible that riparian vegetation assemblages may have been affected in similar ways by land use across the region. Our results imply that even sites within the least disturbed catchments may be affected by distal land uses and overall catchment modification in the region, potentially via macro‐ecological processes, for example, teleconnections (McCluney et al. 2014). Land use intensity and associated changes in land cover especially are also very likely to have contributed, in addition to dams and weirs, to overall modification of flow regimes in the region and, in turn, its effects on riparian vegetation (Mackay et al. 2014).

Research on forest ecology in tropical and subtropical regions is hampered by their sheer complexity and diversity (Pyke et al. 2001). In riparian habitats, the challenges of conducting vegetation surveys are further compounded by the difficulty of acquiring complementary hydrologic and hydraulic data as well as data for other significant environmental variables (e.g., soil type, land use, and clearance history) with which to interpret floristic patterns. As a result, relatively few studies have attempted to relate such a broad suite of potential drivers to riparian vegetation in subtropical catchments. Our results highlight some of the difficulties in determining hydro‐ecological relationships in these landscapes, especially with regard to disentangling the effects of multiple, covarying drivers – in this case, climate and hydrology.

We suggest that for classification to be more useful in the context of ELOHA applications, the development of a subclassification based on geomorphology and stream hydraulics (as proposed by Poff et al. 2010) may assist interpretation of the main environmental influences on riparian communities. For example, the relative tolerances of species to physical disturbances such as shear stresses associated with stream hydraulic conditions would be worthy of further investigation within ELOHA studies involving riparian vegetation. The information gained by incorporating potentially confounding variables into flow–ecology relationships is not part of the ELOHA framework, but has been explored in the fish component of the southeast Queensland study using a multivariate approach (e.g., Arthington et al. 2014). Controlled experiments, including those conducted as part of an adaptive management strategy, may be required, in addition to vegetation surveys, to better inform environmental flow planning in such complex riparian and riverine systems (Poff et al. 2003). A functional, trait‐based approach to understanding vegetation patterns in relation to hydrology, flow classes, stream hydraulics, and other environmental drivers may also prove informative (Burton et al. 2009; Merritt et al. 2010).

This study has demonstrated the relevance of an ELOHA type flow classification to variation in the structure of riparian vegetation across a subtropical landscape, and the importance of studying the influence of covarying drivers on riparian patterns. Our approach guided the development of a conceptual model that distinguishes the relative influence of flow versus other broad‐scale drivers using sites from two contrasting flow classes. We suggest that management of environmental flows to influence riparian vegetation assemblages would have most potential at sites dominated by rheophytic species for which hydrological influences likely override other controls. In contrast, where vegetation assemblages are dominated by a diverse array of typical rainforest species, other factors including broad‐scale climatic gradients and topographic and soil variables have greater influence than hydrology, and the vegetation is likely to be less responsive to environmental flow management.

## Conflict of Interest

None declared.

## Supporting information


**Figure S1.** Box and whisker plots of riparian vegetation metrics across flow classes for rivers of subtropical south east Queensland.
**Figure S2.** Box and whisker plots of abundance of common riparian species (per ha) across flow classes for rivers of subtropical south east Queensland.
**Figure S3.** Box and whisker plots of environmental variables across flow classes for rivers of subtropical south east Queensland.
**Table S1.** List of species recorded, their families and successional stage (assigned according to Kanowski et al. (2010).
**Table S2.** Species indicator values for flow classes.
