# Supplementary material for: Does stream flow structure woody riparian vegetation in subtropical catchments?
Source: Ecol Evol. 2016 Jul 27;6(16):5950–63. doi: 10.1002/ece3.2249 (PMC4983605; doi:10.1002/ece3.2249)

**Supplementary Material**

**Table S1.** List of species recorded, their families and successional stage (assigned according to Kanowski et al. (2010)). For species absent from this database successional stage was assigned based on experience or available literature. If successional stage could not be confidently assigned it was removed from the determination of the successional metrics.

| Species | Code | Family | Origin | Habit | Successional stage |
| --- | --- | --- | --- | --- | --- |
| *Alangium villosum* | Ala_vil | ALANGIACEAE | native | tree | L |
| *Euroschinus falcatus* | Eur_fal | ANACARDIACEAE | native | tree | EM |
| *Rhodosphaera rhodanthema* | Rho_rho | ANACARDIACEAE | native | tree | M |
| *Alyxia ruscifolia* | Aly_rus | APOCYNACEAE | native | shrub | ML |
| *Carissa ovata* | Car_ova | APOCYNACEAE | native | shrub | M |
| *Tabernaemontana pandacaqui* | Tab_pan | APOCYNACEAE | native | shrub | EML |
| *Polyscias elegans* | Pol_ele | ARALIACEAE | native | tree | EM |
| *Schefflera actinophylla* | Sch_act | ARALIACEAE | exotic | tree | M |
| *Araucaria cunninghamii* | Ara_cun | ARAUCARIACEAE | native | tree | EML |
| *Archontophoenix spp* | Arc_spp | ARECACEAE | native | tree | L |
| *Linospadix monostachya* | Lin_mon | ARECACEAE | native | tree | L |
| *Argophyllum nullumense* | Arg_nul | ARGOPHYLLACEAE | native | shrub | ? |
| *Nandina spp* | Nan_spp | BERBERIDACEAE | exotic | shrub | ? |
| *Jacaranda mimosifolia* | Jac_mim | BIGNONIACEAE | exotic | tree | EM |
| *Tecoma capensis* | Tec_cap | BIGNONIACEAE | exotic | shrub | ? |
| *Tecoma stans* | Tec_sta | BIGNONIACEAE | exotic | tree | EM |
| *Canarium australasicum* | Can_aus | BURSERACEAE | native | tree | ML |
| *Senna pendula* | Sen_pen | CAESALPINIACEAE | exotic | shrub | EM |
| *Senna septemtrionalis* | Sen_sep | CAESALPINIACEAE | exotic | shrub | EM |
| *Senna spp.* | Sen_spp | CAESALPINIACEAE | exotic | shrub | EM |
| *Senna sulfurea* | Sen_sul | CAESALPINIACEAE | native | shrub | EM |
| *Capparis arborea* | Cap_arb | CAPPARACEAE | native | shrub | ML |
| *Casuarina cunninghamiana* | Cas_cun | CASUARINACEAE | native | tree | EM |
| *Casuarina littoralis* | Cas_lit | CASUARINACEAE | native | tree | ML |
| *Pleurostylia opposita* | Ple_opp | CELASTRACEAE | native | tree | M |
| *Pseudoweinmannia lachnocarpa* | Pse_lac | CUNONIACEAE | native | tree | L |
| *Cordyline spp* | Cor_spp | DRACAENACEAE | native | shrub | ML |
| *Diospyros australis* | Dio_aus | EBENACEAE | native | tree | ML |
| *Diospyros ellipticifolia* | Dio_ell | EBENACEAE | native | tree | ML |
| *Diospyros fasciculosa* | Dio_fas | EBENACEAE | native | tree | ML |
| *Diospyros geminata* | Dio_gem | EBENACEAE | native | tree | ML |
| *Diospyros pentamera* | Dio_pen | EBENACEAE | native | tree | ML |
| *Diploglottis australis* | Dip_aus | EBENACEAE | native | tree | EML |
| *Elaeocarpus grandis* | Ela_gra | ELAEOCARPACEAE | native | tree | EML |
| *Elaeocarpus obovatus* | Ela_obo | ELAEOCARPACEAE | native | tree | ML |
| *Sloanea australis* | Slo_aus | ELAEOCARPACEAE | native | tree | L |
| *Sloanea woollsii* | Slo_woo | ELAEOCARPACEAE | native | tree | L |
| *Alchornea ilicifolia* | Alc_ili | EUPHORBIACEAE | native | shrub | ML |
| *Breynia oblongifolia* | Bre_obl | EUPHORBIACEAE | native | shrub | EM |
| *Bridelia exaltata* | Bri_exa | EUPHORBIACEAE | native | tree | ML |
| *Bridelia leichhardtii* | Bri_lei | EUPHORBIACEAE | native | tree | ML |
| *Cleistanthus cunninghamii* | Cle_cun | EUPHORBIACEAE | native | tree | ML |
| *Croton acronychioides* | Cro_acr | EUPHORBIACEAE | native | shrub | L |
| *Dissiliaria baloghioides* | Dis_bal | EUPHORBIACEAE | native | tree | ? |
| *Drypetes deplanchei* | Dry_dep | EUPHORBIACEAE | native | tree | ML |
| *Glochidion ferdinandi* | Glo_fer | EUPHORBIACEAE | native | tree | EM |
| *Mallotus claoxyloides* | Mal_cla | EUPHORBIACEAE | native | tree | M |
| *Mallotus discolor* | Mal_dis | EUPHORBIACEAE | native | tree | EM |
| *Mallotus philippensis* | Mal_phi | EUPHORBIACEAE | native | tree | EM |
| *Phyllanthus microcladus* | Phy_mic | EUPHORBIACEAE | native | shrub | ? |
| *Ricinus communis* | Ric_com | EUPHORBIACEAE | exotic | shrub | ? |
| *Eupomatia bennettii* | Eup_ben | EUPOMATIACEAE | native | shrub | ML |
| *Eupomatia laurina* | Eup_lau | EUPOMATIACEAE | native | tree | ML |
| *Castanospermum australe* | Cas_aus | FABACEAE | native | tree | ML |
| *Erythrina species 'Croftby'* | Ery_spe | FABACEAE | native | tree | ? |
| *Leucaena leucocephala* | Leu_leu | FABACEAE | exotic | tree | E |
| *Citronella moorei* | Cit_moo | ICACINACEAE | native | tree | L |
| *Clerodendrum floribundum* | Cle_flo | LAMIACEAE | native | tree | EM |
| *Vitex melicopea* | Vit_mel | LAMIACEAE | native | shrub | ? |
| *Beilschmiedia obtusifolia* | Bei_obt | LAURACEAE | native | tree | ML |
| *Cinnamomum camphora* | Cin_cam | LAURACEAE | exotic | tree | ML |
| *Cinnamomum oliveri* | Cin_oli | LAURACEAE | native | tree | L |
| *Cryptocarya bidwillii* | Cry_bid | LAURACEAE | native | tree | L |
| *Cryptocarya glaucescens* | Cry_gla | LAURACEAE | native | tree | ML |
| *Cryptocarya laevigata* | Cry_lae | LAURACEAE | native | shrub | L |
| *Cryptocarya macdonaldii* | Cry_mac | LAURACEAE | native | tree | L |
| *Cryptocarya obovata* | Cry_obo | LAURACEAE | native | tree | L |
| *Cryptocarya sclerophylla* | Cry_scl | LAURACEAE | native | tree | L |
| *Cryptocarya triplinervis* | Cry_tri | LAURACEAE | native | tree | EML |
| *Endiandra discolor* | End_dis | LAURACEAE | native | tree | L |
| *Endiandra globosa* | End_glo | LAURACEAE | native | tree | L |
| *Endiandra pubens* | End_pub | LAURACEAE | native | tree | L |
| *Endiandra sieberi* | End_sie | LAURACEAE | native | tree | ML |
| *Endiandra virens* | End_vir | LAURACEAE | native | tree | ? |
| *Neolitsea dealbata* | Neo_dea | LAURACEAE | native | tree | ML |
| *Hibiscus heterophyllus* | Hib_het | MALVACEAE | native | shrub | EM |
| *Dysoxylum rufum* | Dys_ruf | MELIACEAE | native | tree | ML |
| *Melia azedarach* | Mel_aze | MELIACEAE | native | tree | M |
| *Synoum glandulosum* | Syn_gla | MELIACEAE | native | tree | ML |
| *Toona ciliata* | Too_cil | MELIACEAE | native | tree | EML |
| *Turraea pubescens* | Tur_pub | MELIACEAE | native | shrub | ML |
| *Acacia bakeri* | Aca_bak | MIMOSACEAE | native | tree | EM |
| *Acacia fimbriata* | Aca_fim | MIMOSACEAE | native | tree | EM |
| *Acacia spp.* | Aca_spp | MIMOSACEAE | native | tree | E |
| *Archidendron muellerianum* | Arc_mue | MIMOSACEAE | native | tree | ML |
| *Pararchidendron pruinosum* | Par_pru | MIMOSACEAE | native | tree | ML |
| *Daphnandra apatela* | Dap_apa | MONIMIACEAE | native | tree | L |
| *Daphnandra tenuipes* | Dap_ten | MONIMIACEAE | native | tree | M |
| *Wilkea huegeliana* | Wil_hue | MONIMIACEAE | native | shrub | ML |
| *Wilkea macrophylla* | Wil_mac | MONIMIACEAE | native | shrub | L |
| *Ficus coronata* | Fic_cor | MORACEAE | native | tree | EM |
| *Ficus fraseri* | Fic_fra | MORACEAE | native | tree | EM |
| *Ficus obliqua* | Fic_obl | MORACEAE | native | tree | L |
| *Ficus opposita* | Fic_opp | MORACEAE | native | tree | ? |
| *Ficus racemosa* | Fic_rac | MORACEAE | native | tree | ? |
| *Ficus virens* | Fic_vir | MORACEAE | native | tree | L |
| *Ficus watkinsiana* | Fic_wat | MORACEAE | native | tree | L |
| *Morus spp* | Mor_spp | MORACEAE | exotic | tree | ? |
| *Streblus brunonianus* | Str_bru | MORACEAE | native | tree | ML |
| *Ardisia crenata* | Ard_cre | MYRSINACEAE | exotic | shrub | L |
| *Myrsine variabilis* | Myr_var | MYRSINACEAE | native | shrub | ML |
| *Angophora spp.* | Ang_spp | MYRTACEAE | native | tree | ? |
| *Archirhodomyrtus beckleri* | Arc_bec | MYRTACEAE | native | tree | ML |
| *Backhousia myrtifolia* | Bac_myr | MYRTACEAE | native | tree | ML |
| *Eucalyptus spp* | Euc_spp | MYRTACEAE | native | tree | EM |
| *Eugenia uniflora* | Eug_uni | MYRTACEAE | exotic | shrub | EM |
| *Lophostemon confertus* | Lop_con | MYRTACEAE | native | tree | EML |
| *Lophostemon spp* | Lop_spp | MYRTACEAE | native | tree | ? |
| *Lophostemon suaveolens* | Lop_sua | MYRTACEAE | native | tree | ? |
| *Melaleuca bracteata* | Mel_bra | MYRTACEAE | native | tree | ? |
| *Melaleuca quinquenervia* | Mel_qui | MYRTACEAE | native | tree | E |
| *Melaleuca salignus* | Cal_sal | MYRTACEAE | native | tree | ML |
| *Melaleuca spp* | Mel_spp | MYRTACEAE | native | tree | ? |
| *Melaleuca viminalis* | Cal_vim | MYRTACEAE | native | tree | EM |
| *Pilidiostigma rhytidosperma* | Pil_rhy | MYRTACEAE | native | tree | ? |
| *Rhodamnia argentea* | Rho_arg | MYRTACEAE | native | tree | ML |
| *Rhodamnia rubescens* | Rho_rub | MYRTACEAE | native | tree | M |
| *Rhodomyrtus psidioides* | Rho_psi | MYRTACEAE | native | tree | EM |
| *Syzygium australe* | Syz_aus | MYRTACEAE | native | tree | ML |
| *Syzygium floribundum* | Syz_flo | MYRTACEAE | native | tree | ML |
| *Syzygium luehmannii* | Syz_lue | MYRTACEAE | native | tree | L |
| *Syzygium oleosum* | Syz_ole | MYRTACEAE | native | tree | ML |
| *Syzygium smithii* | Syz_smi | MYRTACEAE | native | tree | ML |
| *Tristaniopsis laurina* | Tri_lau | MYRTACEAE | native | tree | ML |
| *Ochna serrulata* | Och_ser | OCHNACEAE | exotic | shrub | M |
| *Ligustrum lucidum* | Lig_luc | OLEACEAE | exotic | tree | ML |
| *Ligustrum sinense* | Lig_sin | OLEACEAE | exotic | shrub | ML |
| *Notelaea longifolia* | Not_lon | OLEACEAE | native | tree | M |
| *Notelaea microcarpa* | Not_mic | OLEACEAE | native | shrub | M |
| *Olea paniculata* | Ole_pan | OLEACEAE | native | tree | ML |
| *Auranticarpa rhombifolia* | Aur_rho | PITTOSPORACEAE | native | tree | M |
| *Bursaria incana* | Bur_inc | PITTOSPORACEAE | native | shrub | ? |
| *Hymnosporum flavum* | Hym_fla | PITTOSPORACEAE | native | tree | EM |
| *Pittosporum multiflorum* | Pit_mul | PITTOSPORACEAE | native | tree | ML |
| *Pittosporum undulatum* | Pit_und | PITTOSPORACEAE | native | tree | E |
| *Grevillea robusta* | Gre_rob | PROTEACEAE | native | tree | M |
| *Helicia glabriflora* | Hel_gla | PROTEACEAE | native | tree | ML |
| *Hicksbeachia pinnatifolia* | Hic_pin | PROTEACEAE | native | tree | L |
| *Macadamia tetraphylla* | Mac_tet | PROTEACEAE | native | tree | L |
| *Alphitonia excelsa* | Alp_exc | RHAMNACEAE | native | tree | EM |
| *Prunus spp.* | Pru_spp | ROSACEAE | exotic | tree | ? |
| *Rosaceae fruit tree* | Ros_fru | ROSACEAE | exotic | tree | ? |
| *Atractocarpus chartaceus* | Atr_cha | RUBIACEAE | native | tree | L |
| *Cyclophyllum coprosmoides* | cyc_cop | RUBIACEAE | native | tree | ML |
| *Hodgkinsonia ovatiflora* | Hod_ova | RUBIACEAE | native | tree | ML |
| *Ixora beckleri* | Ixo_bec | RUBIACEAE | native | tree | L |
| *Pavetta australiensis* | Pav_aus | RUBIACEAE | native | shrub | ML |
| *Psychotria daphnoides* | Psy_dap | RUBIACEAE | native | shrub | ML |
| *Psychotria loniceroides* | Psy_lon | RUBIACEAE | native | shrub | ML |
| *Psychotria spp. 'shute harbour'* | Psy_spp | RUBIACEAE | native | shrub | ML |
| *Psydrax odorata* | Psy_odo | RUBIACEAE | native | shrub | ? |
| *Acronychia oblongifolia* | Acr_obl | RUTACEAE | native | tree | ML |
| *Citrus X taitensis* | Cit_X t | RUTACEAE | exotic | shrub | M |
| *Flindersia schottiana* | Fli_sch | RUTACEAE | native | tree | EML |
| *Medicosma cunninghamii* | Med_cun | RUTACEAE | native | tree | L |
| *Micromelum minutum* | Mic_min | RUTACEAE | native | tree | ML |
| *Alectryon tomentosus* | Ale_tom | SAPINDACEAE | native | tree | ML |
| *Arytera distylis* | Ary_dis | SAPINDACEAE | native | tree | L |
| *Arytera divaricata* | Ary_div | SAPINDACEAE | native | tree | L |
| *Atalaya salicifolia* | Ata_sal | SAPINDACEAE | native | tree | ML |
| *Cupaniopsis anacardioides* | Cup_ana | SAPINDACEAE | native | tree | ML |
| *Cupaniopsis newmanii* | cup_new | SAPINDACEAE | native | tree | L |
| *Cupaniopsis serrata* | Cup_ser | SAPINDACEAE | native | tree | L |
| *Elattostachys nervosa* | Ela_ner | SAPINDACEAE | native | tree | L |
| *Elattostachys xylocarpa* | Ela_xyl | SAPINDACEAE | native | tree | L |
| *Guioa semiglauca* | Gui_sem | SAPINDACEAE | native | tree | EM |
| *Jagera pseudorhus* | Jag_pse | SAPINDACEAE | native | tree | EM |
| *Lepiderema pulchella* | lep_pul | SAPINDACEAE | native | tree | L |
| *Mischarytera lautereriana* | Mis_lau | SAPINDACEAE | native | tree | L |
| *Mischocarpus australis* | Mis_aus | SAPINDACEAE | native | tree | ML |
| *Mischocarpus pyriformis* | Mis_pyr | SAPINDACEAE | native | tree | L |
| *Sarcopteryx stipitata* | Sar_sti | SAPINDACEAE | native | tree | ML |
| *Toechima tenax* | Toe_ten | SAPINDACEAE | native | tree | ML |
| *Planchonella australis* | Pla_aus | SAPOTACEAE | native | tree | L |
| *Pouteria queenslandica* | Pou_que | SAPOTACEAE | native | tree | L |
| *Ailanthus triphysa* | Ail_tri | SIMAROUBACEAE | native | tree | ML |
| *Quassia spp.* | Qua_spp | SIMAROUBACEAE | native | shrub | L |
| *Cestrum nocturnum* | Ces_noc | SOLANACEAE | exotic | shrub | ? |
| *Solanum chrysotrichum* | Sol_chr | SOLANACEAE | exotic | shrub | E |
| *Solanum mauritianum* | Sol_mau | SOLANACEAE | exotic | tree | EM |
| *Solanum torvum* | Sol_tor | SOLANACEAE | exotic | shrub | EM |
| [*Argyrodendron trifoliolatum*](http://plantnet.rbgsyd.nsw.gov.au/cgi-bin/NSWfl.pl?page=nswfl&lvl=gn&name=Argyrodendron) | Her_tri | STERCULIACEAE | native | tree | L |
| *Brachychiton spp* | Bra_spp | STERCULIACEAE | native | tree | ML |
| *Commersonia bartramia* | Com_bar | STERCULIACEAE | native | tree | EM |
| *Sterculia quadrifida* | Ste_qua | STERCULIACEAE | native | tree | M |
| *Symplocos spp* | Sym_spp | SYMPLOCACEAE | native | shrub | ML |
| *Wikstroemia indica* | Wik_ind | THYMELAEACEAE | native | shrub | EM |
| *Aphananthe philippinensis* | Aph_phi | ULMACEAE | native | tree | ML |
| *Celtis sinensis* | Cel_sin | ULMACEAE | exotic | tree | EML |
| *Trema tomentosa* | Tre_tom | ULMACEAE | native | tree | EM |
| *Lantana camara* | Lan_cam | VERBENACEAE | exotic | shrub | EM |

**Table S2.** Species indicator values for flow classes. *Exotic species

| Species | Code | Flow class | Bank Full | Near stream |
| --- | --- | --- | --- | --- |
| *Melaleuca bracteata* | Mel_bra | 1 | 60.67 | 30.96 |
| *Castanospermum australe* | Cas_aus | 1 |  | 43.27 |
| *Casuarina cunninghamiana* | Cas_cun | 1 | 36.36 |  |
| *Celtis sinensis** | Cel_sin | 1 |  | 42.25 |
| *Lantana camara** | Lan_cam | 2 | 48.41 |  |
| *Melaleuca viminalis* | Mel_vim | 2 | 46.64 |  |
| *Aphananthe philippinensis* | Aph_phi | 2 | 39.63 |  |
| *Streblus brunonianus* | Str_bru | 3 | 38.38 |  |
| *Tristaniopsis laurina* | Tri_lau | 4 | 66.17 |  |
| *Mallotus claoxyloides* | Mal_cla | 4 | 31.40 |  |
| *Neolitsea dealbata* | Neo_dea | 5 | 74.89 |  |
| *Archontophoenix spp* | Arc_spp | 5 | 73.66 | 50.00 |
| *Cinnamomum oliveri* | Cin_oli | 5 | 70.21 |  |
| *Cinnamomum camphora** | Cin_cam | 5 | 54.82 | 42.85 |
| *Cryptocarya obovata* | Cry_obo | 5 | 50.55 |  |
| *Guioa semiglauca* | Gui_sem | 5 | 50.07 |  |
| *Euroschinus falcatus* | Eur_fal | 5 | 50.00 |  |
| *Sloanea australis* | Slo_aus | 5 | 46.65 |  |
| *Endiandra pubens* | End_pub | 5 | 46.63 |  |
| *Ardisia crenata* | Ard_cre | 5 | 43.06 |  |
| *Diospyros pentamera* | Dio_pen | 5 | 32.20 |  |
| *Wilkea macrophylla* | Wil_mac | 5 | 30.10 |  |

**Figure S1.** Box and whisker plots of riparian vegetation metrics across flow classes for rivers of subtropical south east Queensland. Flow regime characteristics for each flow class are provided in Table 1. Riparian metric descriptions are provided in Table 2.


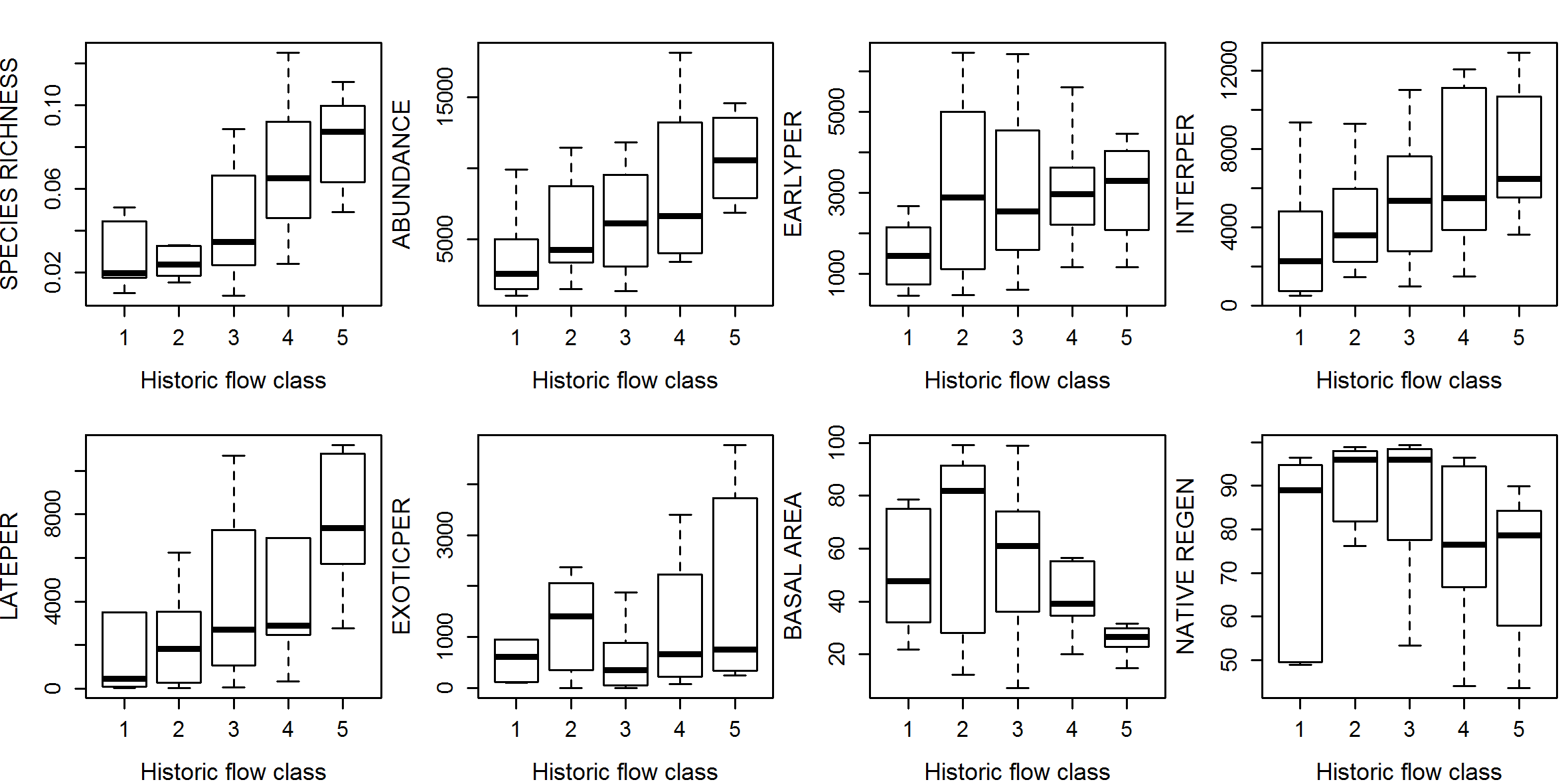


**Figure S2.** Box and whisker plots of abundance of common riparian species (per ha) across flow classes for rivers of subtropical south east Queensland. Flow regime characteristics for each flow class are provided in Table 1. Species codes are given in Table S1*.*

**
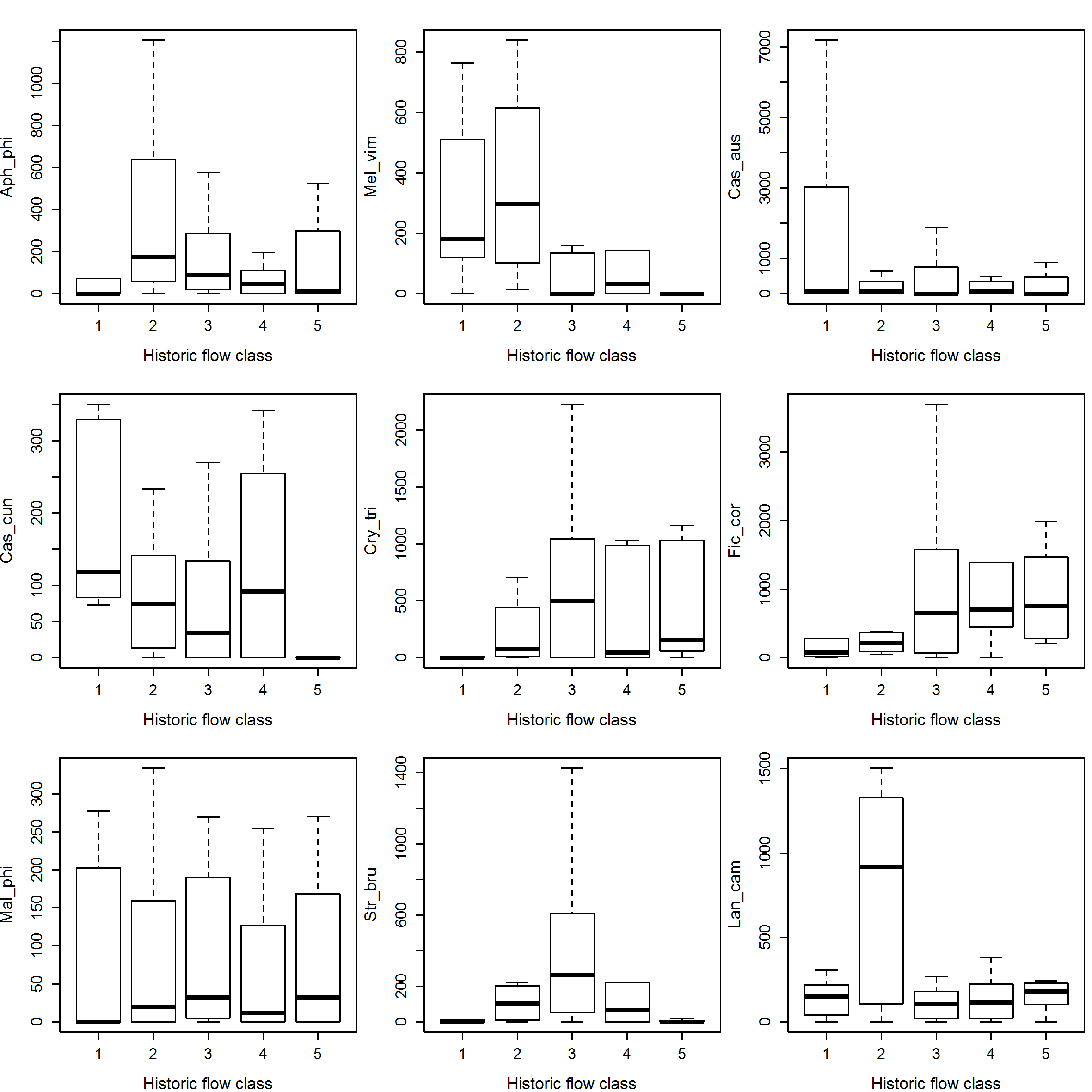
**

**Figure S3.** Box and whisker plots of environmental variables across flow classes for rivers of subtropical south east Queensland. Flow regime characteristics for each flow class are provided in Table 1.


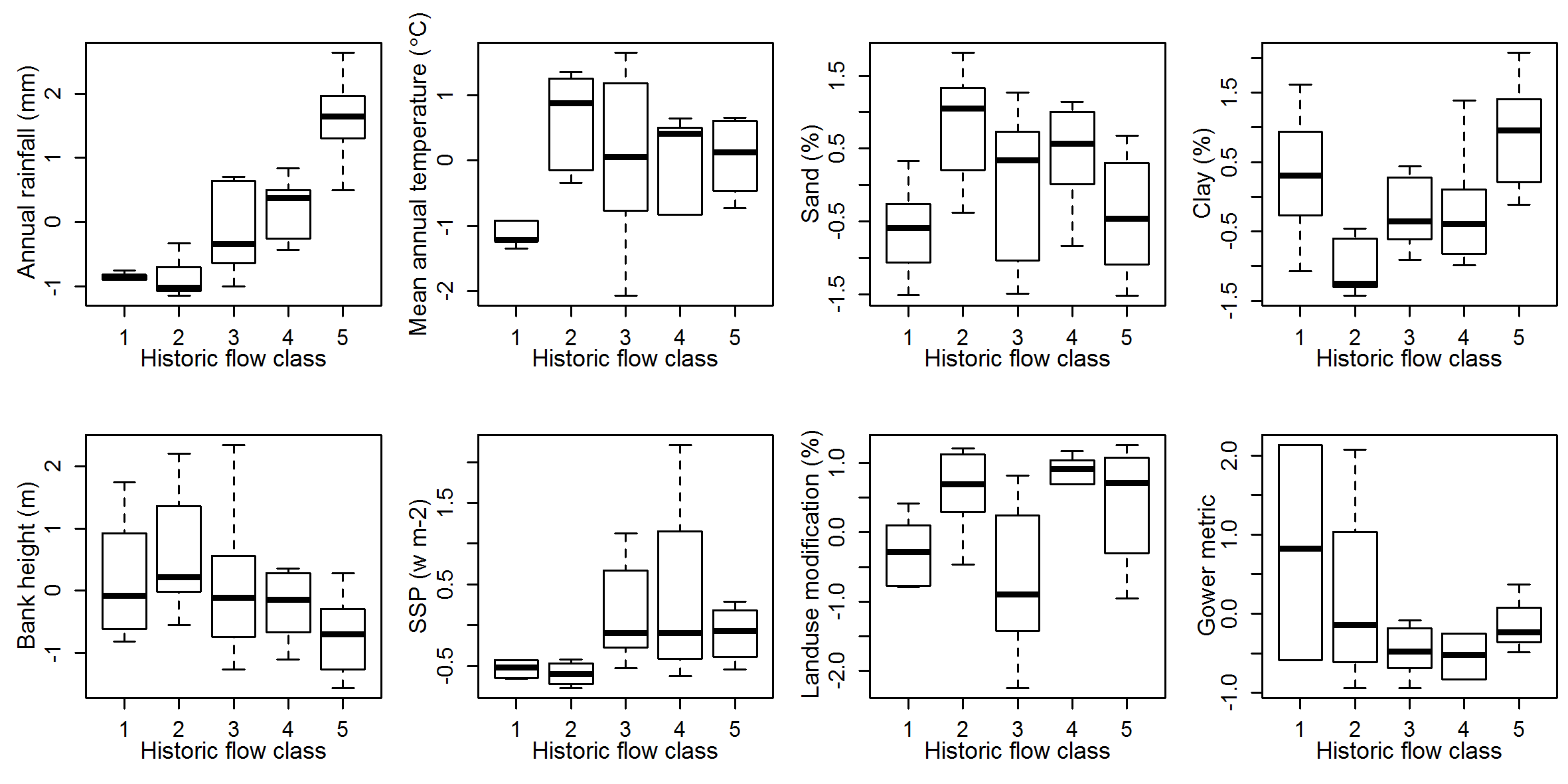

Supplement: Supplementary file 1 — Figure S1. Box and whisker plots of riparian vegetation metrics across flow classes for rivers of subtropical south east Queensland. Figure S2. Box and whisker plots of abundance of common riparian species (per ha) across flow classes for rivers of subtropical south east Queensland. Figure S3. Box and whisker plots of environmental variables across flow classes for rivers of subtropical south east Queensland. Table S1. List of species recorded, their families and successional stage (assigned according to Kanowski et al. (2010). Table S2. Species indicator values for flow classes. [file ECE3-6-5950-s001.docx]
